# *Beet yellows virus* replicase and replicative compartments: parallels with other RNA viruses

**DOI:** 10.3389/fmicb.2013.00038

**Published:** 2013-03-06

**Authors:** Vladimir A. Gushchin, Andrey G. Solovyev, Tatyana N. Erokhina, Sergey Y. Morozov, Alexey A. Agranovsky

**Affiliations:** ^1^Faculty of Biology, Moscow State UniversityMoscow, Russia; ^2^A. N. Belozersky Institute of Physico-Chemical Biology, Moscow State UniversityMoscow, Russia; ^3^M. M. Shemyakin and Y. A. Ovchinnikov Institute of Bioorganic Chemistry, Russian Academy of SciencesMoscow, Russia

**Keywords:** RNA virus replication, membrane vesicles, virus replication factory, endoplasmic reticulum modification, intracellular traffic

## Abstract

In eukaryotic virus systems, infection leads to induction of membranous compartments in which replication occurs. Virus-encoded subunits of the replication complex mediate its interaction with membranes. As replication platforms, RNA viruses use the cytoplasmic surfaces of different membrane compartments, e.g., endoplasmic reticulum (ER), Golgi, endo/lysosomes, mitochondria, chloroplasts, and peroxisomes. *Closterovirus* infections are accompanied by formation of multivesicular complexes from cell membranes of ER or mitochondrial origin. So far the mechanisms for vesicles formation have been obscure. In the replication-associated 1a polyprotein of *Beet yellows virus *(BYV) and other closteroviruses, the region between the methyltransferase and helicase domains (1a central region (CR), 1a CR) is marginally conserved. Computer-assisted analysis predicts several putative membrane-binding domains in the BYV 1a CR. Transient expression of a hydrophobic segment (referred to here as CR-2) of the BYV 1a in *Nicotiana benthamiana* led to reorganization of the ER and formation of ~1-μm mobile globules. We propose that the CR-2 may be involved in the formation of multivesicular complexes in BYV-infected cells. This provides analogy with membrane-associated proteins mediating the build-up of “virus factories” in cells infected with diverse positive-strand RNA viruses (alpha-like viruses, picorna-like viruses, flaviviruses, and nidoviruses) and negative-strand RNA viruses (bunyaviruses).

Eukaryotic viruses from disparate groups, both DNA and RNA containing ones, induce in cells drastic rearrangement of the membranes leading to formation of “virus organelles” or “virus factories”. It is suggested that these compartments protect virus nucleic acids from nucleases and specific cell defense mechanisms, along with creating sufficiently high concentration of interacting templates, replication proteins, and substrates. Recent excellent reviews cover the topic in full (den Boon and Ahlquist, 2010; Netherton and Wileman, 2011; Verchot, 2011). In this work, we attempted to reconcile the ultrastructural data available for several RNA virus groups with our findings of the membrane-modifying activity of a hydrophobic segment of the 1a polyprotein of beet yellows closterovirus (BYV).

## OPEN ULTRASTRUCTURES: BUNYAVIRUSES

*Bunyamwera virus* (BunV) is an enveloped virus with a negative-sense RNA genome (~12 kb) divided among three segments. In infected mammalian cells, BunV infection leads to formation of tubular structures (up to 50 per cell) encompassing the Golgi membranes, actin, myosin I, and viral non-structural protein NSm (Fontana et al., 2008). The tubes are in close contact with mitochondria and rough endoplasmic reticulum (ER), possibly serving as sources of host factors (e.g., translation elongation factor eEF-2 and ribosomal proteins) aiding the virus replication. Transcription and replication of BunV occur inside the “globular domain,” a U-like structure at one end of the tubes. The replicative complexes consisting of BunV nucleoproteins and RNA replicase, concentrate on the inner surface of the globular domain. BunV transcription yields mRNAs that are transferred to rough ER for translation, and replication produces the progeny nucleoproteins transported to the Golgi stacks modified by inserted BunV surface glycoproteins, for particle maturation (Fontana et al., 2008).

The model by Fontana et al. (2008) implies dynamic changes of, and communication between, the cell membranous compartments induced by bunyavirus infection, driven mainly by actin filaments and that the viral NSm. Apparently, the primary transcription of the gene encoding NSm must occur prior to changes in Golgi. The BunV replication-associated globular domains are open structures, unlike the vesicles and spherules induced by positive-sense RNA viruses (see below). This might reflect a nuclease-protected state of the BunV genomic and antigenomic RNA templates, the absence of dsRNA (which might trigger RNA interference in cells) in negative-sense RNA viruses replication, and employment of strategies against host defense mechanisms (Léonard et al., 2006; Habjan et al., 2008).

## “CLOSED” ULTRASTRUCTURES: NIDOVIRUSES

Nidoviruses are enveloped viruses with positive-sense RNA genomes of 13–16 kb (arteriviruses) and ~30 kb (coronaviruses). The replication-associated proteins are encoded in overlapping 5′-open reading frames (ORFs) 1a and 1b, and translation of the genomic RNA yields polyproteins 1a and 1ab autocatalytically processed into non-structural proteins forming the replication complex (reviewed in Gorbalenya, 2008). Using ER membranes as the main source, nidoviruses induce in cells double-membrane vesicles (DMVs, 150–300 nm in diameter), convoluted membranes (CMs), and vesicle packets (VPs) of merged DMVs. These structures accumulate dsRNA and replication-associated proteins. The coronavirus nsp3, nsp4, and nsp6 encompass transmembrane domains and are plausibly the key factors for membrane remodeling. Recent EM tomography analysis of the severe acute respiratory syndrome (SARS) virus-infected cells allowed refinement of the topology of SARS ultrastructures (Knoops et al., 2008). DMVs and VPs apparently form a network with connections to each other and to the ER; however, no openings to the cytosol were detected (Knoops et al., 2008). The apparently “closed” state of the DMV network poses a yet unresolved question as to how the coronavirus factory exchanges ribonucleotide triphosphates (rNTP) substrates and newly synthesized RNA with the cytosol (Knoops et al., 2008).

Picornaviruses, small non-enveloped viruses with (+)RNA genome of ~8 kb, induce heterogeneous (50–500 nm) DMVs of the ER, Golgi and lysosomal origin (Bienz et al., 1990; Schlegel et al., 1996). Some commonality of the picornavirus and coronavirus ultrastructures (particularly, the absence of apparent bridges to cytosol) has been noted (den Boon and Ahlquist, 2010; Netherton and Wileman, 2011). However, the question of whether picornaviruses indeed produce a “closed” network of DMVs awaits further study.

## ULTRASTRUCTURES WITH NECKS: ALPHA-LIKE VIRUSES, NODAVIRUSES, FLAVIVIRUSES

The alpha-like superfamily unites positive-sense RNA viruses of animals (alphaviruses, rubiviruses, hepeviruses), and plants (e.g., bromoviruses, tobamoviruses, tymoviruses), whose genomes encode the conserved domains of methyltransferase (MTR), NTPase/helicase (HEL), and RNA polymerase (POL; Goldbach, 1987). The replication system of Brome mosaic virus (BMV) has been studied in considerable detail. BMV has a tripartite genome (~8.2 kb), with RNA-1 and RNA-2 coding, respectively, for proteins 1a (MTR–HEL) and 2a (POL). Early in infection, 1a binds to perinuclear ER membranes via an amphipathic helix located in non-conserved region between the MTR and HEL (Liu et al., 2009). It should be noted parenthetically that in the capping enzyme of Semliki Forest alphavirus, the equivalent membrane-binding function is governed by an unrelated amphipathic helix within the MTR (Ahola et al., 1999). The BMV 1a protein causes membrane invaginations and engages 2a^Pol^ and viral RNA templates (rendering them non-sensitive to nucleases) to the membrane (den Boon and Ahlquist, 2010). Each mature vesicle retains a thin neck (~8 nm) to cytosol. The vesicle encompasses hundreds of 1a molecules forming inner layer, 10–20 2a^Pol^ molecules, and a few molecules of genomic and antigenomic RNAs (Schwartz et al., 2002). Other alpha-like viruses (with the exception of closteroviruses, see Section 5 of this paper) apparently induce morphologically similar ultrastructures, the line-up of 50–100 nm single-membrane vesicles, often with detectable necks to cytosol, originating from endosomes and lysosomes (alphaviruses), ER (tobamoviruses), tonoplasts (alfamoviruses), and chloroplasts (tymoviruses; reviewed in Netherton and Wileman, 2011; Verchot, 2011).

Flock house nodavirus (FHV) has compact bipartite (+)RNA genome (~4.5 kb). RNA-1 encodes protein A, a multifunctional RNA replicase (Venter and Schneemann, 2008). The replicase molecules, via the N-terminal mitochondrial targeting signal and transmembrane domain, attach to the outer mitochondrial membrane and cause its invaginations, thus producing numerous 50-nm vesicles (spherules) with 10-nm necks into cytosol (Kopek et al., 2007). The interior of the vesicles is lined by ~100 copies of replicase (Kopek et al., 2007). Hence, FHV and BMV, albeit distantly related evolutionarily, employ similar mechanisms of membranes modification and replication factory build-up.

Dengue flavivirus (DenV) is an enveloped virus with a monopartite (+)RNA genome (~11 kb) encoding a single polypeptide precursor (Bartenschlager and Miller, 2008). Non-structural proteins NS2A, NS4A, and NS4B bear transmembrane domains and are responsible for transformation of ER membranes into a network of interconnected VPs (~90-nm single-membrane vesicles surrounded by common membrane), CVs, and virion budding sites (Welsch et al., 2009). The VPs retain dsRNA and viral replication proteins. Noteworthy, the DenV-induced network has ~8-nm neck-like openings to the cytosol (Welsch et al., 2009). Hence, the flavivirus factory combines features of the coronavirus network and the *bromovirus* and nodavirus necked ultrastructures.

## INTRACELLULAR TRANSPORT OF REPLICATION COMPLEXES

After entry of one or a few virus particles or viral nucleic acid molecules into the cell, these must move to the compartments where genome expression and replication proceed. The intracellular transport of viral particles and replication complexes is rather an active process than mere diffusion, as cytosol is a highly viscous matter where translocation of molecules or complexes exceeding a ~500-kDa limit is impeded (Luby-Phelps, 2000; Greber and Way, 2006). Microinjection of fluorescently labeled tobacco mosaic virus (TMV) RNA into tobacco trichome cells rapidly leads to formation of granules associated with the ER, that are translocated along the actin network (Christensen et al., 2009). Using TMV particles where RNA and coat protein were labeled with different fluorescent dyes, it was found that that both signals initially co-localized on the same granules, indicating that the virus may become attached to the ER/actin prior to uncoating (Christensen et al., 2009).

There is emerging evidence that the replication complexes and/or the associated membranous ultrastructures of (+)RNA viruses are transported along the cytoskeleton. Thus, the replication factories of turnip mosaic potyvirus (TuMV) are represented by heterogeneous vesicles of 0.6 to 4.3 μm in diameter accumulating in the perinuclear zone. Interestingly, some vesicles are highly motile with an average velocity of 0.45 μm/s. Their movement is unidirectional and occurs in “stop and go” mode (Cotton et al., 2009; Grangeon et al., 2010, 2012). Likewise, the distribution of *tobamovirus* replication-associated complexes in cells is dynamic and cytoskeleton-dependent (Más and Beachy, 1999; Szecsi et al., 1999). The *tobamovirus* 126-kDa (MTR–HEL) protein and the 126-kDa-induced vesicles bind to and traffic along the actin microfilaments (Liu et al., 2005). In the hepatitis C flavivirus system, interaction of two replication proteins, NS3 (RNA HEL – serine proteinase) and NS5A (phosphoprotein), provides for binding and movement of the replication complex along microtubules and actin filaments (Lai et al., 2008). Mouse *norovirus* appears to utilize microtubules during the early stages of replication to establish localization of the replicative complexes proximal to the microtubule organizing center (Hyde et al., 2012). There is a significant overlap in the function and regulation of microtubule and actin networks in animal and plant systems (Goode et al., 2000; Barton and Overall, 2010; Sampathkumar et al., 2011). Many proteins, including molecular motors, have been demonstrated to associate with both networks to coordinate intracellular trafficking and movement of organelles (Petrásek and Schwarzerová, 2009; Viklund et al., 2009; Mucha et al., 2011; Meiri et al., 2012). A number of disparate viruses, including Semliki forest virus, vaccinia virus, and respiratory syncytial virus, have been shown to utilize, in a coordinated manner, both the microtubule and actin networks to facilitate replication (Newsome et al., 2004; Kallewaard et al., 2005; Spuul et al., 2011).

Plant viruses often utilize cytoskeleton for the cell-to-cell movement (Harries et al., 2009, 2010). The movement proteins interact with replication complexes as well as with actin microfilaments and microtubules (Grangeon et al., 2012; Solovyev et al., 2012; Tilsner et al., 2012). Both cytoskeletal systems may act as conduits for individual viral RNAs, transported ribonucleoproteins, as well as large replication complexes to reach plasmodesmata and thus to assist intercellular trafficking (Bamunusinghe et al., 2009; Harries et al., 2010; Schoelz et al., 2011; Grangeon et al., 2012; Pena and Heinlein, 2012; Solovyev et al., 2012; Tilsner and Oparka, 2012; Tilsner et al., 2012). These data indicate that diverse (+)RNA viruses of plants may use cytoskeleton for intracellular trafficking of replication complexes or the components thereof, to plasmodesmata.

## MULTIVESICULAR COMPLEXES OF CLOSTEROVIRUSES

**FIGURE 1:**
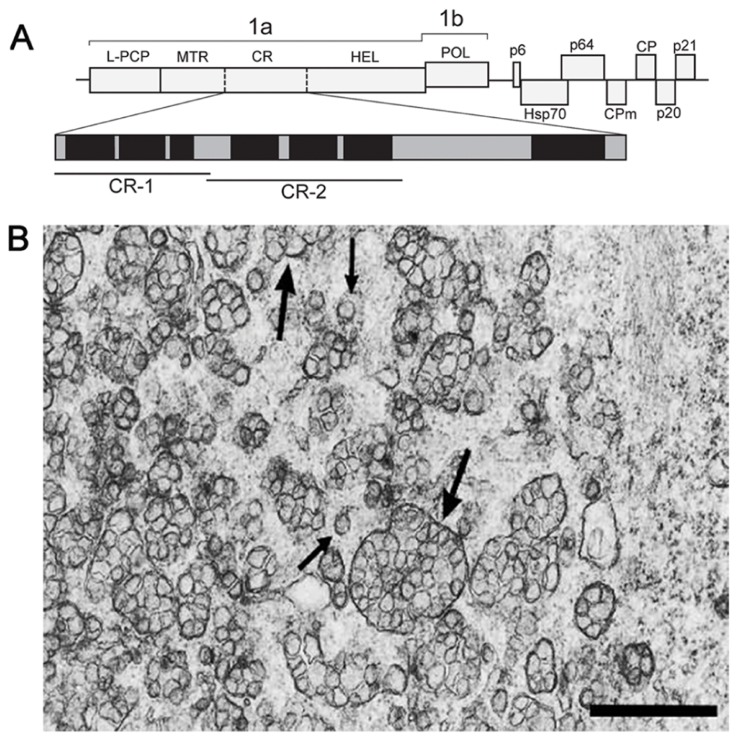
**(A)** Schematic representation of the BYV genome and the encoded proteins. 1a polyprotein encompasses the leader papain-like cysteine proteinase (L-PCP), methyltransferase (MTR), unique central region (CR), and NTPase/helicase (HEL). Vertical solid bar, established cleavage site of the L-PCP; broken bars, arbitrary cleavage sites within the 1a polyprotein calculated from the apparent molecular masses of the MTR- and HEL-containing proteins. Enlarged map of hydrophobic domains (black) and hydrophilic hinges (gray) in the 1a CR is shown below. The portions used for cloning and expression as GFP C-terminal fusions (CR-1 and CR-2) are indicated by bars. **(B)** Electron microscopy of the BYV-induced ultrastructures in leaf parenchyma cells of *Tetragonia expansa* (21 days p.i.). Double-membrane vesiles (DMVs, small arrows) and vesicle packets (VPs, large arrows) on a tissue section embedded in Epon after fixation with glutaraldehyde and OsO_4_. Scale bar, 1 μm.

Members of the *Closteroviridae* family are related to alpha-like viruses with respect to conservation of key replication-associated protein domains (MTR–HEL–POL), but strikingly resemble nidoviruses in the genome size, layout, and expression pattern (Agranovsky, 1996; Karasev, 2000; Dolja et al., 2006). The beet yellows closterovirus (BYV) 15.5-kb genome encodes the replication-associated proteins in 5′-proximal ORFs 1a and 1b (**Figure 1A**). Translation of these ORFs is expected to yield NH_2_-coterminal 1a and 1ab polyproteins encompassing, respectively, the arrays of papain-like cysteine proteinase (PCP)–MTR–central region(CR)–HEL and PCP–MTR–CR–HEL–POL (L-PCP, leader PCP domain; CR, non-conserved CR; **Figure 1A**; Agranovsky et al., 1994). The autocatalytic cleavage of BYV polyproteins by the PCP at Gly^588^/Gly^589^ releases the 66-kDa leader protein (Zinovkin et al., 2003) which activates amplification of the BYV RNA (Peremyslov et al., 1998; Peng and Dolja, 2000). The 1a and 1ab polyproteins are further processed by a yet unknown proteolytic activity(-ies) into at least three fragments, of which the 63-kDa MTR-containing and 100-kDa HEL-containing proteins were identified in infected plants (Erokhina et al., 2000). The ~70-kDa protein(s) corresponding to the 1a CR (**Figure 1A**) has not been yet detected.

In plant cells, closteroviruses induce the formation of ~100-nm DMVs and multivesicular complexes (single-membrane vesicles surrounded by a common membrane; **Figure 1B**; Cronshaw et al., 1966; Esau et al., 1967; Esau and Hoefert, 1971; Lesemann, 1988). The multivesicular complexes often neighbor with stacks of aligned filamentous BYV particles (Cronshaw et al., 1966; Esau et al., 1967). These ultrastructures broadly resemble the DMVs and VPs produced by nidoviruses and flaviviruses (see Sections 2 and 3 in this paper), and are referred to here as DMVs and VPs for simplicity. The BYV replication-associated proteins (L-PCP, MTR, and HEL) co-localize with the DMV and VP membranes, supporting the role of these ultrastructures as replication platforms (Erokhina et al., 2001; Zinovkin et al., 2003). The membranes in *closterovirus* DMVs and VPs are likely to be derived from ER (*Crinivirus*; Wang et al., 2010) or mitochondria (*Ampelovirus*; Kim et al., 1989; Faoro et al., 1992; Faoro and Carzaniga, 1995). Whether these ultrastructures have “closed” or “necked” state, remains unknown.

**FIGURE 2:**
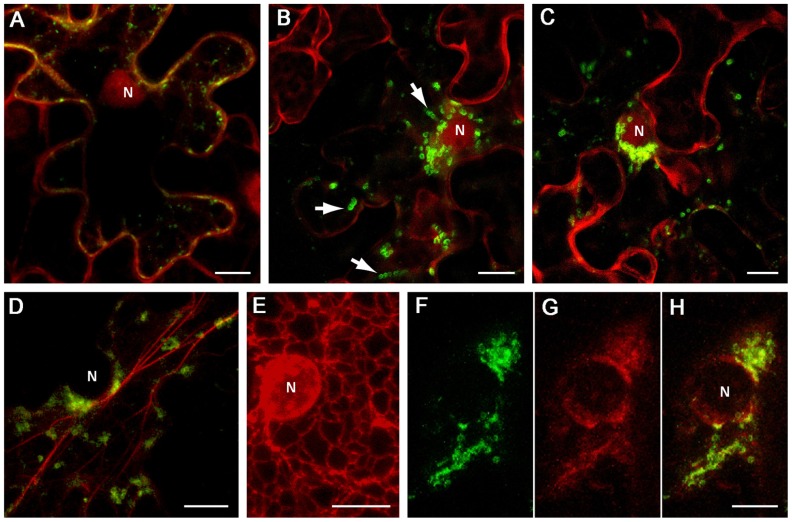
**Localization of GFP-fused CR-1 and CR-2 in epidermal cells of *N. benthamiana* leaves**. Proteins were expressed by agroinfiltration and visualized at 48 h post infiltration by confocal laser scanning microscopy. (**A)** Co-expression of GFP:CR-1 with the red fluorescent marker protein mCherry, which localizes to the cytoplasm and the nucleoplasm in plant cells (Lee et al., 2008). **(B)** and **(C) **Co-expression of GFP:CR-2 with mCherry in two individual cells. Arrows indicate the motile CR-2 globules revealed in frame captures. **(D)** Co-expression of GFP-CR-2 with YFP-Tal (red channel), a fluorescent marker for actin cytoskeleton (Shemyakina et al., 2011). **(E) **Expression of ER-mRFP, the protein targeted to the ER lumen by N-terminal signal peptide and C-terminal ER-retention signal (Haseloff et al., 1997), in the perinuclear region of a plant cell. (**F–H**) Co-expression of GFP:CR-2 with ER-mRFP. **(F)** Perinuclear groups of GFP:CR-2-containing globules. **(G)** Modified perinuclear ER representing diffuse membrane reservoirs. **(H)** Overlap of images **(F)** and **(G)**. All images represent the superpositions of series of confocal optical sections. N, nucleus. Scale bar, 10 μm.

Inspection of the BYV 1a CR sequence (approximately aa 1100 to 1800; **Figure 1A**) using hydropathicity plot drawing software (protScale; Kyte and Doolittle, 1982) revealed several hydrophobic stretches longer than 20 aa forming putative alpha helixes, which resembled membrane-binding domains. Two segments of the 1a CR predicted to form separate hydrophobic domains, CR-1 (aa 1114–1301), and CR-2 (aa 1301–1498; **Figure 1A**), were cloned as green fluorescent protein (GFP) fusions in a binary vector. Upon transient expression in *Nicotiana benthamiana* leaves the fusions showed distinct distribution of the fluorescence. The GFP:CR-1 produced aggregates of heterogeneous shape and size (0.2–1 μm, average 0.5 μm) accumulated at the cell periphery (**Figure 2A**), whereas the GFP:CR-2-induced uniform globules ~1 μm in diameter mostly concentrated around the nucleus (**Figures 2B,C**). Some CR-2-induced globules were apparently motile (**Figure 2B**). Further, we found that the CR-2 globules co-localized with actin filaments (**Figure 2D**), suggesting that the globules might be translocated along the actin network. In cells expressing the GFP:CR-2, the ER network transformed into diffuse membrane reservoirs partially co-localized with the perinuclear groups of GFP:CR-2 globules (cf. **Figures 2E,F–H**). These data corroborate the recent findings by Bryce Falk and colleagues for lettuce infectious yellows virus (genus *Crinivirus* of the *Closteroviridae*), i.e., the rearrangement of perinuclear ER in *N. tabacum* protoplasts inoculated with LIYV RNA1 transcripts, specifically the R1-322 transcript encoding only the 1a and 1ab replicative proteins (Wang et al., 2010).

With due caution in interpreting the results presented in **Figure 2**, it is tempting to speculate that the phenotypes induced by the BYV CR-2 segment might reflect the formation of BYV replication-associated ultrastructures. It is possible that the build-up of *closterovirus* replication platforms depends on the ER membranes and is accompanied by essential changes in perinuclear ER, and that the BYV 1a protein contains a membrane anchor (CR-2) in the region between MTR and HEL, as is the case with BMV 1a protein (Liu et al., 2009). Further study is necessary to elucidate the fine structure of the BYV CR-2-induced globules and their relationship to DMVs and VPs produced in naturally infected cells, as well as to verify the significance of the actin network in transport of the *closterovirus* factory components within the cell.

## Conflict of Interest Statement

The authors declare that the research was conducted in the absence of any commercial or financial relationships that could be construed as a potential conflict of interest.

## References

[B1] Agranovsky A. A. (1996). Principles of molecular organization, expression and evolution of closteroviruses: over the barriers. *Adv. Virus Res.* 47 119–158 8895832 10.1016/S0065-3527(08)60735-6 PMC7130501

[B2] Agranovsky A. A. Koonin E. V. Boyko V. P. Maiss E. Froetschl R. Lunina N. A.(1994). Beet yellows closterovirus: complete genome structure and identification of a leader papain-like thiol protease. *Virology* 198 311–324 8259666 10.1006/viro.1994.1034

[B3] Ahola T. Lampio A. Auvinen P Kääriäinen L. (1999). Semliki Forest virus mRNA capping enzyme requires association with anionic membrane phospholipids for activity. *EMBO J.* 18 3164–3172 10357827 10.1093/emboj/18.11.3164 PMC1171397

[B4] Bamunusinghe D. Hemenway C. L. Nelson R. S. Sanderfoot A. A. Ye C. M. Silva M. A.(2009). Analysis of potato virus X replicase and TGBp3 subcellular locations. *Virology* 393 272–285 19729179 10.1016/j.virol.2009.08.002

[B5] Bartenschlager R. Miller S. (2008). Molecular aspects of Dengue virus replication. *Future Microbiol.* 3 155–165 18366336 10.2217/17460913.3.2.155

[B6] Barton D. A. Overall R. L. (2010). Cryofixation rapidly preserves cytoskeletal arrays of leaf epidermal cells revealing microtubule co-alignments between neighbouring cells and adjacent actin and microtubule bundles in the cortex. *J. Microsc.* 237 79–88 20055921 10.1111/j.1365-2818.2009.03305.x

[B7] Bienz K. Egger D. Troxler M. Pasamontes L. (1990). Structural organization of poliovirus RNA replication is mediated by viral proteins of the P2 genomic region. *J. Virol.* 64 1156–1163 2154600 10.1128/jvi.64.3.1156-1163.1990 PMC249229

[B8] Christensen N. Tilsner J. Bell K. Hammann P. Parton R. Lacomme C.(2009). The 5^′^ cap of tobacco mosaic virus (TMV) is required for virion attachment to the actin/endoplasmic reticulum network during early infection. *Traffic* 10 536–551 19220815 10.1111/j.1600-0854.2009.00889.x

[B9] Cotton S. Grangeon R. Thivierge K. Mathieu I. Ide C. Wei T.(2009). Turnip mosaic virus RNA replication complex vesicles are mobile, align with microfilaments, and are each derived from a single viral genome. *J. Virol.* 83 10460–10471 19656892 10.1128/JVI.00819-09 PMC2753101

[B10] Cronshaw J. Hoefert L. L. Esau K. (1966). Ultrastructural features of Beta leaves infected with beet yellows virus. *J. Cell Biol.* 31 429–443 5971643 10.1083/jcb.31.3.429 PMC2107074

[B11] den Boon J. A. Ahlquist P. (2010). Organelle-like membrane compartmentalization of positive-strand RNA virus replication factories. *Annu. Rev. Microbiol.* 64 241–256 20825348 10.1146/annurev.micro.112408.134012

[B12] Dolja V. V. Kreuze J. F. Valkonen J. P. (2006). Comparative and functional genomics of closteroviruses. *Virus Res.* 117 38–51 16529837 10.1016/j.virusres.2006.02.002 PMC7172929

[B13] Fontana J. López-Montero N. Elliott R. M. Fernández J. J. Risco C. (2008). The unique architecture of *Bunyamwera virus* factories around the Golgi complex. *Cell. Microbiol.* 10 2012–2028 18547336 10.1111/j.1462-5822.2008.01184.x PMC7162186

[B14] Erokhina T. N. Vitushkina M. V. Zinovkin R. A. Lesemann D. E. Jelkmann W. Koonin E. V.(2001). Ultrastructural localisation and epitope mapping of beet yellows closterovirus methyltransferase-like and helicase-like proteins. *J. Gen. Virol.* 82 1983–1994 11458006 10.1099/0022-1317-82-8-1983

[B15] Erokhina T. N. Zinovkin R. A. Vitushkina M. V. Jelkmann W. Agranovsky A. A. (2000). Detection of beet yellows closterovirus methyltransferase-like and helicase-like proteins in vivo using monoclonal antibodies. *J. Gen. Virol.* 81 597–603 10675397 10.1099/0022-1317-81-3-597

[B16] Esau K. Cronshaw J. Hoefert L. L. (1967). Relation of beet yellows virus to the phloem and to movement in the sieve tube. *J. Cell Biol.* 32 71–87 10976202 10.1083/jcb.32.1.71 PMC2107099

[B17] Esau K. Hoefert L. L. (1971). Cytology of beet yellows virus infection in Tetragonia. I. Parenchyma cells in infected leaf. *Protoplasma* 72 255–273 10.1007/BF01286411 5112776

[B18] Faoro F. Carzaniga R. (1995). Cyotchemistry and immuno-cytochemistry of the inclusion bodies induced by grapevine leafroll-associated closteroviruses GLRaV-1 and GLRaV-3. *Rivista di Patologia Vegetale* 5 85–94

[B19] Faoro F. Tornaghi R. Cinquanta S. Belli G. (1992). Cytopathology of leafroll-associated virus III (GLRaV-III). *Rivista di Patologia Vegetale* 2 67–83

[B20] Grangeon R. Cotton S Laliberté J. F. (2010). A model for the biogenesis of turnip mosaic virus replication factories. *Commun. Integr. Biol.* 3 363–365 20798828 10.4161/cib.3.4.11968 PMC2928320

[B21] Grangeon R. Jiang J Laliberté J. F. (2012). Host endomembrane recruitment for plant RNA virus replication. *Curr. Opin. Virol.* 2 683–690 23123078 10.1016/j.coviro.2012.10.003 PMC7185485

[B22] Greber U. F. Way M. (2006). A superhighway to virus infection. *Cell* 124 741–754 16497585 10.1016/j.cell.2006.02.018

[B23] Goldbach R. (1987). Genome similarities between plant and animal RNA viruses. *Microbiol. Sci.* 4 197–202 3153611

[B24] Goode B. L. Drubin D. G. Barnes G. (2000). Functional cooperation between the microtubule and actin cytoskeletons. *Curr. Opin. Cell Biol.* 12 63–71 10679357 10.1016/s0955-0674(99)00058-7

[B25] Gorbalenya A. E. (2008). “Genomics and evolution of the Nidovirales,” in *Nidoviruses*, eds Perlman S. Gallagher T. Snijder E. J. (Washington: ASM Press) 15–28

[B26] Habjan M. Andersson I. Klingstróm J. Schümann M. Martin A. Zimmermann P.(2008). Processing of genome 5^′^ termini as a strategy of negative-strand RNA viruses to avoid RIG-I-dependent interferon induction. *PLoS ONE* 3:e2032 10.1371/journal.pone.0002032 PMC2323571 18446221

[B27] Harries P. A. Park J. W. Sasaki N. Ballard K. D. Maule A. J. Nelson R. S. (2009). Differing requirements for actin and myosin by plant viruses for sustained intercellular movement. *Proc. Natl. Acad. Sci. U.S.A.* 41 17594–17599 19805075 10.1073/pnas.0909239106 PMC2753639

[B28] Harries P. A. Schoelz J. E. Nelson R. S. (2010). Intracellular transport of viruses and their components: utilizing the cytoskeleton and membrane highways. *Mol. Plant Microbe Interact.* 11 1381–1393 20653412 10.1094/MPMI-05-10-0121

[B29] Haseloff J. Siemering K. R. Prasher D. C. Hodge S. (1997). Removal of a cryptic intron and subcellular localization of green fluorescent protein are required to mark transgenic *Arabidopsis* plants brightly. *Proc. Natl. Acad. Sci. U.S.A.* 94 2122–2127 9122158 10.1073/pnas.94.6.2122 PMC20051

[B30] Hyde J. L. Gillespie L. K. Mackenzie J. M. (2012). Mouse norovirus 1 utilizes the cytoskeleton network to establish localization of the replication complex proximal to the microtubule organizing center. *J. Virol.* 86 4110–4122 22301146 10.1128/JVI.05784-11 PMC3318650

[B31] Kallewaard N. L. Bowen A. L. Crowe J. E. Jr. (2005). Cooperativity of actin and microtubule elements during replication of respiratory syncytial virus. *Virology* 331 73–81 15582654 10.1016/j.virol.2004.10.010

[B32] Karasev A. V. (2000). Genetic diversity and evolution of closteroviruses. *Annu. Rev. Phytopathol*. 38 293–324 11701845 10.1146/annurev.phyto.38.1.293

[B33] Kim K. S. Gonsalves D. Teliz D. Lee K. W. (1989). Ultrastructure and mitochondrial vesiculation associated with closterovirus-like particles in leafroll-diseased grapevines. *Phytopathology* 79 357–360

[B34] Knoops K. Kikkert M. Worm S. H. Zevenhoven-Dobbe J. C. van der Meer Y. Koster A. J.(2008). SARS coronavirus replication is supported by a reticulovesicular network of modified endoplasmic reticulum. *PLoS Biol.* 6:e226 10.1371/journal.pbio.0060226 PMC2535663 18798692

[B35] Kopek B. G. Perkins G. Miller D. J. Ellisman M. H. Ahlquist P. (2007). Three – dimensional analysis of a viral RNA replication complex reveals a virus-induced mini-organelle. *PLoS Biol.* 5:e220 10.1371/journal.pbio.0050220 PMC1945040 17696647

[B36] Kyte J. Doolittle R. F. (1982). A simple method for displaying the hydropathic character of a protein. *J. Mol. Biol.* 157 105–132 7108955 10.1016/0022-2836(82)90515-0

[B37] Lai C. K. Jeng K. S. Machida K Lai M. M. C. (2008). Association of Hepatitis C virus replication complexes with microtubules and actin filaments is dependent on the interaction of NS3 and NS5A. *J. Virol.* 82 8838–8848 18562541 10.1128/JVI.00398-08 PMC2519648

[B38] Lee L. Y. Fang M. J. Kuang L. Y. Gelvin S. B. (2008). Vectors for multi-color bimolecular fluorescence complementation to investigate protein-protein interactions in living plant cells. *Plant Methods* 4 24 10.1186/1746-4811-4-24 PMC2572157 18922163

[B39] Léonard V. H. Kohl A. Hart T. J. Elliott R. M. (2006). Interaction of Bunyamwera Orthobunyavirus NSs protein with mediator protein MED8: a mechanism for inhibiting the interferon response. *J. Virol.* 80 9667–9675 16973571 10.1128/JVI.00822-06 PMC1617248

[B40] Lesemann D. E. (1988). “Cytopathology”. in *The Plant Viruses,* ed.Milne R. G. (New York: Plenum) vol. 4 179–235

[B41] Liu J. Z. Blancaflor E. B. Nelson R. S. (2005). The tobacco mosaic virus 126-kilodalton protein, a constituent of the virus replication complex, alone of within the complex aligns with and traffics along microfilaments. *Plant Physiol.* 138 1853–1865 16040646 10.1104/pp.105.065722 PMC1183377

[B42] Liu L. Westler W. M. den Boon J. A. Wang X. Diaz A. Steinberg H. A.(2009). An amphipathic α-helix controls multiple roles of Brome mosaic virus protein 1a in RNA replication complex assembly and function. *PLoS Pathog. * 5:e1000351 10.1371/journal.ppat.1000351 PMC2654722 19325881

[B43] Luby-Phelps K. (2000). Cytoarchitecture and physical properties of cytoplasm: volume, viscosity, diffusion, intracellular surface area. *Int. Rev. Cytol.* 192 189–221 10553280 10.1016/s0074-7696(08)60527-6

[B44] Más P. Beachy R. N. (1999). Replication of tobacco mosaic virus on endoplasmic reticulum and role of the cytoskeleton and virus movement protein in intracellular distribution of viral RNA. *J. Cell Biol.* 147 945–958 10579716 10.1083/jcb.147.5.945 PMC2169346

[B45] Meiri D. Marshall C. B. Greeve M. A. Kim B. Balan M. Suarez F.(2012). Mechanistic insight into the microtubule and actin cytoskeleton coupling through dynein-dependent RhoGEF inhibition. *Mol. Cell* 9 642–655 22405273 10.1016/j.molcel.2012.01.027

[B46] Mucha E. Fricke I. Schaefer A. Wittinghofer A. Berken A. (2011). Rho proteins of plants – functional cycle and regulation of cytoskeletal dynamics. *Eur. J. Cell Biol.* 90 934–943 21277045 10.1016/j.ejcb.2010.11.009

[B47] Netherton C. L. Wileman T. (2011). Virus factories, double membrane vesicles and viroplasm generated in animal cells. *Curr. Opin. Virol.* 1 381–387 22440839 10.1016/j.coviro.2011.09.008 PMC7102809

[B48] Newsome T. P. Scaplehorn N. Way M. (2004). SRC mediates a switch from microtubule- to actin-based motility of vaccinia virus. *Science* 306 124–129 15297625 10.1126/science.1101509

[B49] Pena E. J. Heinlein M. (2012). RNA transport during TMV cell-to-cellmovement. *Front. Plant Sci. * 3:193 10.3389/fpls.2012.00193 PMC3428586 22973280

[B50] Peng C. W. Dolja V. V. (2000). Leader proteinase of the beet yellows closterovirus: mutation analysis of the function in genome amplification. *J. Virol.* 74 9766–9770 11000252 10.1128/jvi.74.20.9766-9770.2000 PMC112412

[B51] Peremyslov V. V. Hagiwara Y. Dolja V. V. (1998). Genes required for replication of the 15.5*-*kilobase RNA genome of a plant *closterovirus*. *J. Virol.* 72 5870–5876 9621048 10.1128/jvi.72.7.5870-5876.1998 PMC110390

[B52] Petrásek J Schwarzerová K. (2009). Actin and microtubule cytoskeleton interactions. *Curr. Opin. Plant Biol.* 12 728–734 19854097 10.1016/j.pbi.2009.09.010

[B53] Sampathkumar A. Lindeboom J. J. Debolt S. Gutierrez R. Ehrhardt D. W. Ketelaar T.(2011). Live cell imaging reveals structural associations between the actin and microtubule cytoskeleton in *Arabidopsis*. *Plant Cell* 23 2302–2313 21693695 10.1105/tpc.111.087940 PMC3160026

[B54] Schlegel A. Giddings T. H. Jr. Ladinsky M. S. Kirkegaard K. (1996). Cellular origin and ultrastructure of membranes induced during poliovirus infection. *J. Virol.* 70 6576–6588 8794292 10.1128/jvi.70.10.6576-6588.1996 PMC190698

[B55] Schoelz J. E. Harries P. A. Nelson R. S. (2011). Intracellular transport of plant viruses: finding the door out of the cell. *Mol. Plant* 4 813–831 21896501 10.1093/mp/ssr070 PMC3183398

[B56] Schwartz M. Chen J. Janda M. Sullivan M. den Boon J. Ahlquist P. (2002). A positive-strand RNA virus replication complex parallels form and function of retrovirus capsids. *Mol. Cell* 9 505–514 11931759 10.1016/s1097-2765(02)00474-4

[B57] Shemyakina E. A. Solovyev A. G. Leonova O. G. Popenko V. I. Schiemann J. Morozov S. Y. (2011). The role of microtubule association in plasmodesmal targeting of Potato mop-top virus movement protein TGBp1. *Open Virol. J*. 5 1–11 21660184 10.2174/1874357901105010001 PMC3109696

[B58] Solovyev A. G. Kalinina N. O. Morozov S. Y. (2012). Recent advances in research of plant virus movement mediated by triple gene block. *Front. Plant Sci. * 3:276 10.3389/fpls.2012.00276 PMC3520053 23248633

[B59] Spuul P. Balistreri G. Hellström K. Golubtsov A. V. Jokitalo E. Ahola T. (2011). Assembly of alphavirus replication complexes from RNA and protein components in a novel trans-replication system in mammalian cells. *J. Virol.* 85 4739–4751 21389137 10.1128/JVI.00085-11 PMC3126202

[B60] Szecsi J. Ding X. S. Lim C. O. Bendahmane M. Cho M. J. Nelson R. S.(1999). Development of tobacco mosaic virus infection sites in *Nicotiana benthamiana*. *Mol. Plant Microbe Interact.* 12 143–152

[B61] Tilsner J. Oparka K. J. (2012). Missing links? – the connection between replication and movement of plant RNA viruses. *Curr. Opin. Virol.* 2 699–705 23036608 10.1016/j.coviro.2012.09.007

[B62] Tilsner J. Linnik O. Wright K. M. Bell K. Roberts A. G. Lacomme C.(2012). The TGB1 movement protein of Potato virus X reorganizes actin and endomembranes into the X-body, a viral replication factory. *Plant Physiol.* 158 1359–1370 22253256 10.1104/pp.111.189605 PMC3291258

[B63] Venter P. A. Schneemann A. (2008). Recent insights into the biology and biomedical applications of Flock House virus. *Cell. Mol. Life Sci.* 65 2675–2687 18516498 10.1007/s00018-008-8037-y PMC2536769

[B64] Verchot J. (2011). Wrapping membranes around plant virus infection. *Curr. Opin. Virol.* 1 388–395 22440840 10.1016/j.coviro.2011.09.009

[B65] Viklund I. M. Aspenström P. Meas-Yedid V. Zhang B. Kopec J. Agren D.(2009). WAFL, a new protein involved in regulation of early endocytic transport at the intersection of actin and microtubule dynamics. *Exp. Cell Res.* 315 1040–1052 19121306 10.1016/j.yexcr.2008.12.004

[B66] Wang J. Stewart L. R. Kiss Z. Falk B. W. (2010). Lettuce infectious yellows virus (LIYV) RNA 1-encoded P34 is an RNA-binding protein and exhibits perinuclear localization. *Virology* 403 67–77 20447670 10.1016/j.virol.2010.04.006

[B67] Welsch S. Miller S. Romero-Brey I. Merz A. Bleck C. K. Walther P.(2009). Composition and threedimensional architecture of the dengue virus replication and assembly sites. *Cell Host Microbe* 5 365–375 19380115 10.1016/j.chom.2009.03.007 PMC7103389

[B68] Zinovkin R. A. Erokhina T. N. Lesemann D. E. Jelkmann W. Agranovsky A. A. (2003). Processing and subcellular localization of the leader papain-like proteinase of beet yellows closterovirus. *J. Gen. Virol.* 84 2265–2270 12867660 10.1099/vir.0.19151-0

